# Translation, cross-cultural adaptation and validation of the English Lequesne Algofunctional index in to Bengali

**DOI:** 10.1186/s12955-020-01583-x

**Published:** 2020-10-19

**Authors:** Tarek Mahmood, Minhaj Rahim Choudhury, Md Nazrul Islam, Syed Atiqul Haq, Md Abu Shahin, Syed Mohammad Monowar Ali, Shamim Ahmed, Md. Nahiduzzamane Shazzad, A. T. M. Tanveer Hasan, Mohammad Abul Kalam Azad, Johannes Jacobus Rasker

**Affiliations:** 1 Department of Medicine, Patuakhali Medical College, Patuakhali, Bangladesh; 2 Department of Rheumatology, Bangabandhu Sheikh Mujib Medical University (BSMMU), Dhaka, Bangladesh; 3 Department of Medicine, Shaheed M. Monsur Ali Medical College, Sirajgonj, Bangladesh; 4 Department of Rheumatology, Enam Medical College and Hospital, Savar, Dhaka, Bangladesh; 5 Department of Psychology, Health and Technology, Faculty of Behavioural Sciences, University of Twente, Enschede, The Netherlands

**Keywords:** Lequesne Algofunctional index, Bengali, Translation, Cross-cultural adaptation, Validation

## Abstract

**Background:**

This study was focused on translation and cultural adaptation of the English Lequesne Algofunctional index (LAI) into Bengali for patients with primary knee osteoarthritis (OA) and testing reliability and validity of the Bengali version of the LAI.

**Methods:**

This study was carried out in the Department of Rheumatology, BSM Medical University, Dhaka, Bangladesh. Using the forward–backward method the English LAI was translated into Bengali including cultural adaptation. For pretesting, A sample of 40 patients with primary knee osteoarthritis were screened using the Bengali version of LAI. Following the pretest, 130 consecutive patients with symptomatic knee OA completed the interviewer administered Bengali LAI, the validated Bengali version of SF-36, Visual Analogue Scale for Pain, Distance Walked and Activities of Daily Living. For the retest 60 randomly selected patients from the cohort were administered the Bengali LAI 7 days later. An item by item analysis was performed. Internal consistency was assessed by Cronbach’s alpha, test–retest reliability by intraclass correlation coefficient (ICC) and Kappa coefficient, construct validity was measured using the Spearman rank correlation coefficient.

**Results:**

It took 3.25 ± 0.71 min to complete the Bengali LAI and the mean score was 9.23 ± 4.58. For the Bengali LAI Cronbach’s alpha score was 0.88, test–retest reliability assessed by ICC was 0.97. For construct validity, excellent convergent validity was achieved (ρ = 0.93) but the divergent validity was moderate (ρ = 0.43).

**Conclusions:**

The Bengali LAI showed excellent convergent validity, internal consistency and test–retest reliability, only the divergent validity was moderate. So, the Bengali LAI can be applied as a HRQoL assessment tool for primary knee OA patients.

## Background

Osteoarthritis is a common chronic, non-communicable disease. The knee is the most frequently affected joint in osteoarthritis (OA) causing pain, reduced mobility and restricting activities of daily living and quality of life [1]. Symptomatic knee OA is more common in women than in men and its prevalence increases with age [1]. In Bangladesh the point prevalence of knee OA is around 10% [2]. There is little or no correlation of radiographic OA changes with clinical symptoms, so therapeutic decisions depend on the pain intensity and physical disability suffered by these patients [1,3].

The measurement of Health Related Quality of Life (HRQoL) is increasingly being used in health care, also for chronic, non-communicable diseases [4]. Several specific instruments are in use for the assessment of osteoarthritis, such as the WOMAC [5] (Western Ontario and McMaster Universities Osteoarthritis Index), KOOS [6] (knee Osteoarthritis Outcome Score) and Lequesne Algofunctional Index (LAI) [7]. None of these instruments can be used as the gold standard due to a lack of unique features. Sun et al. from their study recommended that WOMAC and LAI are acceptable as evaluation tools for primary knee OA [8].

The Lequesne Algofunctional index has an interview format questionnaire, including 10 questions divided into three sections regarding pain, maximum distance walked and activities of daily living [7]. The score ranges from 0 (no pain, no disability) to 24 (maximum pain and disability [7,9]. This is a quite simple index that usually does not cause misunderstanding and takes only 3 to 4 min to complete. The psychometric properties of this index have been studied [10], translated and validated in different countries such as Turkey [11], Korea [12], and Singapore [13], Switzerland [14], France [15].

Bengali is the sixth most widely spoken language in the world with nearly 300 million users [16]. In 2050, it is estimated that the Bengali speaking population will be nearly 400 million [17]. In 2016 there were 163 million people in Bangladesh of which 25% were above 40 years, at an age where the prevalence of knee OA increases [2]. In this context this study was aimed to translate the English LAI into Bengali with cross-cultural adaptation and validation, so that it can be applied in studies among Bengali speaking patients with primary knee OA.

## Materials and methods

### Study design

This was a two-phased study. In phase-I translation and cross-cultural adaptation of English Lequesne Algo-functional index was done into Bengali and in phase-II psychometric properties of the scale were evaluated in Bengali speaking primary knee OA patients.

### Study subjects

In phase I in order to check that the LAI was easily understood, we tested the index on 10 boys and girls in year class six (approximate age 12 years old). A total of 40 patients of both sexes with symptomatic primary knee osteoarthritis, fulfilling the clinical criteria of the American College of Rheumatology (ACR) [18] attending the rheumatology outpatient department (OPD) of BSMMU were enrolled for pretest. In phase-II, for test–retest reliability and validity 130 consecutive patients with symptomatic primary knee OA were studied [18]. Patients having other disabling lower limb osteoarthropathy or myopathy, severely ill patients, patients suffering from severe psychiatric disorders and mentally or physically disabled persons were excluded from the study.

### The scales

The Lequesne Algofunctional Index is a 10-question interview format questionnaire that was designed as a single unit. These 10 parameters are divided in three sections to assess ‘pain or discomfort’ (L1), ‘maximum distance walked’ (L2), and ‘activities of daily living’ (L3). The first section contains five parameters, ‘Pain or discomfort during nocturnal bed rest’ (L1A), ‘duration of morning stiffness or pain after getting up’ (L1B), ‘Remaining standing for 30 min increases pain’ (L1C), ‘Pain on walking’ (L1D), ‘Pain or discomfort when getting up from sitting position without the help of arms’ (L1E). Parameter three and five are graded as 0 = no, 1 = yes. Parameter one is graded as ‘0 = no’, ‘1 = only on movement or in certain positions’, ‘2 = without movement’, parameter two was graded as, ‘0 = no’, ‘1 = less than 15 min’, ‘2 = 15 min or more’, and parameter four was graded as ‘0 = no’, ‘1 = only after walking some distance’, ‘2 = early after starting’. The second section is graded from 0 = unlimited to 6 = less than 100 m, 1 = more than 1 km, but limited, 2 = about 1 km (about 15 min), 3 = from 500 to 900 m (about 8–15 min), 4 = from 300 to 500 m, 5 = from 100 to 300 m. This score is upgraded by one point if the patient uses one walking stick or crutch or by two points if the patient uses two walking sticks or crutches. In the third section parameters are: ‘can you go up a standard flight of stairs’ (L3A), ‘can you go down a standard flight of stairs’ (L3B), ‘can you squat’ (L3C), and ‘can you walk on uneven ground’ (L3D). The score for each parameter is graded as ‘0 = without difficulty’,’0.5 = with slight difficulty’, ‘1 = moderate difficulty’, ‘1.5 = great difficulty’ ‘2 = unable to do’. Though there is a variable number of parameters in the different sections, each section scores minimum 0 to maximum 8. Thus, The Lequesne Algofunctional Index score ranges from 0 to 24 [7].

Other tools in this study were: the Bengali version of SF 36 [19,20], visual analogue scales (0–100 mm) for pain (VAS P), for distance walked (VAS DW), and for activities of daily living (VAS ADL) to assess the construct validity.

### Phase-I: translation and cross-cultural adaptation

The forward backward method is a five-stage process as follows [21]. Stage 1 was the forward translation of the English LAI into Bangla by two native Bengali translators. One of the translators was aware of the concepts of the questionnaire (T1), the other translator was totally blind about the concepts to be measured in this study (T2). In stage 2, the synthesized version of the LAI was developed, two translators and recording observer sat together to synthesize a new version of translation (Ts). In stage 3, the Ts version of the questionnaire was back translated into English by two translators (BT1 and BT2) with good command of the English language, who were totally blind to the original version. In stage 4, an expert committee reviewed the whole proceeding. The committee comprised methodologists, health professionals, language professionals, rheumatologists and the translators (forward and back translators). Materials at disposal of the committee were—LAI English questionnaire, two forward Bengali translations of LAI (T1, T2), synthesized Bengali version of LAI (Ts), two backward translations of the synthesized Bengali version of LAI (BT1, BT2) and corresponding reports. The committee reviewed and compared all the translations and the LAI questionnaire. They verified the semantic, idiomatic, experiential and conceptual equivalence between the English and Bengali version. Consensus was reached on the items and when necessary the translation and back-translation process was repeated to clarify how another wording of an item can work. Whereas the original LAI in English is constructed as statements to be given certain values by the recipient, this is not always suitable for Bengali patients. Some of the original parameters were transposed into a question format. After the completion of the above process, a version of Bengali LAI was developed. This was then given to 10 students in a year class six (age approximately 12 years old) from the point of view of easy comprehension. The questionnaire that had been developed thus far was then tested on 40 Bengali patients with primary knee OA. Each patient was then interviewed to find out what he or she had understood by what was meant in each question and why they had responded the way they did. In this way we were able to hone the Bengal LAI in order to retain the equivalence of the original English version. The distribution of responses was examined to look for a high proportion of missing items or single responses. In this way, the Bengali version of Lequesne Algofunctional Index was developed.

### Phase II: assessment of psychometric properties of Bengali version of Lequesne Algofunctional Index:

After the translation and adaptation process, it is important that the new version contains the measurement properties needed for practical application. A total of 130 consecutive patients with primary knee OA fulfilling the clinical criteria of the ACR [18] were recruited for the study from the outpatient department of Rheumatology, BSMMU. Informed written consent was taken in front of an attendant. After collecting patient’s demographic and clinical characteristics, the Bengali version of LAI, the validated Bengali version SF-36 (Medical Outcomes Study-36 Item Short Form), VAS P, VAS DW, VAS ADL (Visual Analogue Scale for Pain, Distance walked, Activity of Daily Living respectively) were administered. All the VAS used ranged from 0 (no pain, no limitation, no difficulty) to 100 mm (maximum pain, maximum limitation, maximum difficulty). Randomly selected 60 stable patients were requested to complete the Bengali LAI questionnaire after 7 days. No intervention was suggested during that period while the existing medication continued.

### Statistical analysis

Data were analyzed using SPSS17.0 (IBM Statistics Armonk NY USA). All tests were two tailed and were conducted at a 5% level of significance. Internal consistency was assessed by using Cronbach’s alpha and, test–retest reliability using intraclass correlation coefficient (ICC) and kappa coefficient for binary variables [22]. Cronbach’s alpha ≥ 0.7 is generally regarded as acceptable for group comparisons, and ≥ 0.9 for individual comparisons, while an ICC ≥ 0.7 is also considered as acceptable. [23]. Dimensionality was assessed by performing principal component factor analysis with varimax rotation. An eigen value criterion of 1.0 was used and the percentage of variances explained by the principal factor was given [24].

Two types of validity were assessed: content and construct validity. Content validity was assessed by three experts in Rheumatology. Each expert rated each item as either 1 (agreed), 0 (undetermined), or − 1 (disagreed). The index of content validity (ICV) of each item was calculated using summation of scores from each expert divided by the number of experts. Construct validity was assessed using Spearman’s rank correlation coefficient. The construct validity is an important criterion of a questionnaire in which researchers use a measure as an index of a variable that is not itself directly observable [25]. To evaluate construct validity convergent and divergent validities were assessed by observing correlation with theoretically similar and dissimilar parameters. We hypothesized moderate to strong correlations between LAI score and SF-36 physical function domain, LAI pain and stiffness with VAS P, LAI distance walked with VAS DW, LAI activities of daily living with VAS ADL resulting in four a priori hypotheses for convergent construct validity, namely: 1. LAI global score with physical function of SF 36, 2. LAI pain and stiffness with VAS P 3. LAI distance walked with VAS DW and 4. LAI activities of daily living with VAS ADL. In contrast, for discriminant construct validity we hypothesized weak correlations between domains measuring dissimilar constructs, resulting in another four a priori hypotheses, namely: the weak correlations of all three section scores and global score of LAI with SF-36 mental health. Correlation coefficients of > 0.50, 0.30–0.50, and < 0.30 were considered strong, moderate, and weak, respectively [26].

## Results

### Phase-I: translation and cross-cultural adaptation

All the parameters and responses of English LAI were kept in the Bengali version and only minor cultural adaptations were needed. Considering the nuance of Bengali language and people’s responses, parameters were converted to a questionnaire format and taking into account the literacy or comprehension of the Bangladeshi patients terms such as ‘impossible’ were translated into ‘cannot do at all’. The children understood the Bengali LAI without difficulty. The Bengali version of Lequesne Algofunctional index was well understood and accepted by Bengali speaking primary knee OA patients. They agreed that the parameters and findings were important for their disease evaluation and there was no offensive question.

### Phase-II: assessment of psychometric properties of Bengali version of Lequesne Algofunctional Index

#### Characteristics of subjects

The mean age of the participants was 54.7 ± 6.3, among them 60% between 50–54-years of age. The characteristics of the subjects are shown in Table 1.

**Table 1:** Characteristics of subjects completing Bengali LAI

		Number		Percent	
Age: mean ± SD (54.7 ± 6.3)	50–54	78		60.0	
	55–59	26		20.0	
	60–64	11		8.4	
	≥ 65	15		11.5	
Gender	Male	60		46.2	
	Female	70		53.8	
Literacy	Capable of reading and writing	6		4.6	
	None/illiterate	21		16.2	
	Primary	41		31.5	
	Secondary	52		40	
	Bachelor/masters	10		7.7	
Occupation	Housewife	61		46.9	
	Govt. service	8		6.2	
	Private service	15		11.5	
	Businessman	12		9.2	
	Farmer	8		6.2	
	Others	26		20	
Residency	Rural	70		53.8	
	Urban	60		46.2	

#### Item analysis

It took 3.25 ± 0.71 min (minimum 2–maximum 5) to complete the Bengali LAI. The mean score was 9.23 ± 4.58, with minimum 2 and maximum 20. In the composite score of LAI there was no floor and no ceiling effect and there were no missing items. Even in sections, only L2 (maximum distance walked) and L1 (pain and discomfort) showed 27.7% floor effect and 4.6% ceiling effect respectively (Table 2).

**Table 2:** Distribution and reliability of all three sections and global score of LAI

Scales	Mean (SD)	Median (Interquartile range)	Percent at floor/celling	Cronbach’s alpha	Test–retest (ICC)
Pain or discomfort	4.58 (1.72)	5 (1–8)	0/4.6	0.66	0.89
Maximum distance walked	1.48 (1.44)	1 (0–7)	27.7/0		0.97
Activities of daily living	3.18 (1.80)	3 (1–7)	0/0	0.91	0.96
LAI global index	9.23 (4.58)	8.75 (2–20)	0/0	0.88	0.97

#### Reliability of the Bengali LAI

The internal consistency was acceptable as Cronbach’s alpha exceeded the cutoff value of 0.70 recommended for group comparisons, being 0.91, and 0.88 for activities of daily living, and LAI global score respectively. The test–retest reliability was acceptable with the ICC exceeding the cutoff value of 0.7 for all sections as well as the global index (Table 2). ICC and kappa coefficient for test–retest reliability of all the items were acceptable (Table 3).

**Table 3:** Test–retest reliability: intraclass correlation or kappa coefficient for each parameter of the Bengali LAI

Parameters	Intraclass correlation co-efficient (ICC)	Kappa co-efficient
L1A (pain or discomfort during nocturnal bed rest)	0.89	
L1B (duration of morning stiffness or pain after getting up)	0.93	
L1C (remaining standing for 30 min increases pain)	–	0.64
L1D (pain on walking)	0.81	
L1E (pain or discomfort when getting up from sitting position without the help of arms)	–	0.65
L2 (maximum distance walked)	0.97	
L3A (can you go up a standard flight of stairs)	0.90	
L3B (can you go down a standard flight of stairs)	0.92	
L3C (can you squat)	0.91	
L3D (can you walk on uneven ground)	0.93	

Item to scale correlations are displayed in Table 4 for all items except maximum distance walked where there is only one item. All the items of activities of daily living demonstrated acceptable item to scale correlation.

**Table 4:** Item to scale correlation with each section and LAI total

Parameters	Non-parametric Spearman’s rank Correlation (ρ)	
	Sections	LAI
	Pain or discomfort	0.66
L1A (pain or discomfort during nocturnal bed rest)	0.38	0.49
L1B (duration of morning stiffness or pain after getting up)	0.50	0.66
L1C (remaining standing for 30 min increases pain)	0.10	0.26
L1D (pain on walking)	0.25	0.37
L1E (pain or discomfort when getting up from sitting position without the help of arms)	0.16	0.36
	Maximum distance walked	0.67
	Activities of daily living	0.77
L3A (can you go up a standard flight of stairs)	0.62	0.70
L3B (can you go down a standard flight of stairs)	0.75	0.78
L3C (can you squat)	0.66	0.70
L3D (can you walk on uneven ground)	0.70	0.74

#### Construct validity

The construct validity was investigated in two ways. The convergent validity was demonstrated by the presence of expected correlations between global score of LAI and score of the physical functioning domain of SF-36 which was ρ = 0.93. Section score of 1, 2, 3 was correlated with score of VASP (ρ = 0.89), VASDW (ρ = 0.88) and VASADL (ρ = 0.90) respectively (Table 5). The divergent validity was assessed by correlating the global score of LAI and section scores of L1, L2, and L3 with SF-36 mental health score. All sections and the global score of LAI showed weak correlation with SF-36 mental health (Table 5).

**Table 5:** Construct validity of the Bengali Lequesne Algofunctional index

Convergent validity	Spearman correlation (ρ)					
	VASP	VAS DW	VAS ADL	SF 36		
				Physical Function	Pain	Role Physical
L1-pain or discomfort	0.89				0.83	0.82
L2-maximum distance walked		0.88			0.83	0.85
L3-activities of daily living			0.90		0.88	0.90
LG- global score of LAI				0.93	0.89	0.91
*Divergent validity*						
	L1	L2	L3	LG		
Mental health score of SF 36	0.36	0.43	0.44	0.43		

*LAI* Lequesne Algofunctional index, *SF 36* Medical Outcome Score: 36 item short form, *VAS P* visual analogue scale for pain, *VAS DW* visual analogue scale for distance walked, *VAS ADL* visual analogue scale for activities of daily living

Correlation matrix of functional indices [Spearman rank correlation (ρ)]

### Factor analysis

Two factors were found to have retained eigen values > 1. The cumulative percent variance of these factors was 67.8. Table 6 shows the loading of each parameter after varimax rotation on the two factors. However, the clinical parameters of these factors could not be characterized.

**Table 6:** Factors in factor analysis and Varimax rotated factor matrix of the Lequesne index

Factor	F1	F2
*Factors in factor analysis*		
Eigen value	5.6	1.2
% of variance	56.1	11.7
Cumulative %	56.1	67.8
*Varimax rotated factor matrix*		
L1A (pain or discomfort during nocturnal bed rest)	0.71	0.11
L1B (duration of morning stiffness or pain after getting up)	0.82	0.20
L1C (remaining standing for 30 min increases pain)	-0.09	0.84
L1D (pain on walking)	0.33	0.62
L1E (pain or discomfort when getting up from sitting position without the help of arms)	0.69	− 0.19
L2-maximum distance walked	0.66	0.56
L3A (can you go up a standard flight of stairs)	0.66	0.58
L3B (can you go down a standard flight of stairs)	0.76	0.46
L3C (can you squat)	0.75	0.41
L3D (can you walk on uneven ground)	0.77	0.43

Extraction method: principal component analysis

Rotation method: varimax with kaiser normalization

## Discussion

In this two-phased study the English Lequesne Algofunctional Index was translated and culturally adapted in Bengali and then the psychometric properties of this index were evaluated in a clinical setting. There were no culturally unfamiliar parameters in the questionnaire, but minor changes were made maintaining the expression but making it more comprehensible in Bengali. One striking observation during the pretest was keeping or not keeping the target knee joint in the variables, especially in ‘pain and discomfort’ section. Asking response without mentioning the target joint, the participants often gave information based on other affected parts of the body, forgetting the initial instruction. To solve this problem the target knee joint was added to all the variables of ‘pain and discomfort’ section. All the parameters and findings of English LAI were kept in the Bengali version. The Bengali version of Lequesne Algofunctional index was well understood, there was no offensive question.

Internal consistency was assessed by Cronbach’s alpha which was 0.88 for Bengali LAI but a lower Cronbach’s alpha was observed for ‘pain and discomfort’ section. Similar findings had been reported by Xie et al. [13] and Stucki et al. [14]. This section adopted dichotomous response options for two parameters and variable response options for remaining three parameters. Having varying grading options for the different parameters might be responsible for lack of internal consistency, compared with higher internal consistency in the activities of daily living section, where grading of all four items were consistent. Test–retest reliability was assessed by ICC which was 0.97 for LAI global score. The reliability of Bengali LAI is comparable with the Singapore English version [13], the French version [15], better than the Singapore Chinese version [13], the German [14] and Korean versions [12]. It should be noted that, between the item and scale analysis, the Lequesne index was developed as a single score, rather than three individual scales. The three parameters of Sect. 1 did not show the expected correlation within the section and among them one did not show adequate correlation with the scale. However, all the parameters showed better correlation with the global index rather than the individual section. All four parameters of Sect. 3 showed acceptable correlation with the section and scale. It should be noted that unlike section one this section has uniformly distributed response options. Similar findings were observed in the Singapore English and Chinese versions [13].

The construct validity is an important criterion of a questionnaire because no irrefutable gold standard currently exists to assess pain and function in knee OA [8]. To evaluate construct validity convergent and divergent validities were assessed. The convergent validity was assessed by correlating the composite score of the LAI with the score of the physical functioning domain of SF-36 which was ρ = 0.93. Section score of L1, L2, L3 was correlated with score of VASP (ρ = 0.89), VASDW (ρ = 0.88) and VASADL (ρ = 0.90) respectively. But L1, L2, L3 sections also showed excellent correlation with physical functioning domain of SF 36. It might be due to its development as algo-functional index that pain and function parameters address the same tasks. A weak correlation of the global score of LAI and all of its sections were observed with the mental score of SF 36, which implies that it does not hold a strong divergent validity. It might be explained by emotional upset by physical pain and disability. It should also be noted that the average mental health score for SF 36 for knee OA patients or for general population of Bangladesh is not known. The construct validity of this study was totally opposite to a French study, where divergent validity was strong but convergent validity was weak however they used score of anxiety, score of depression, score of Kellgren, and circumference of the thigh for divergent validity [15].

This study also demonstrated that Lequesne index is not an uni-dimensional HRQoL questionnaire, as two factors are found to have retained eigen values > 1. Similar findings were found in Singapore English and Chinese versions [13], where two factors were extracted and in a French study [15] where three factors retained eigen value more than one. Loading of parameters in Sects. 2 and 3 were nearly uniform but all five parameters of section one loaded highly on either factor.

## Conclusions

The main objective of this study was to develop a Bengali version of LAI for the evaluation of quality of life in patients suffering from primary knee OA. Following a standard procedure, a Bengali version of LAI was developed. This Bengali LAI showed excellent internal consistency and test–retest reliability. It showed excellent convergent validity and acceptable divergent validity. So, this Bengali version of LAI may be used for the evaluation of quality of life in Bengali speaking patients suffering from primary knee OA.

## Data Availability

Data is available on reasonable request.
